# Relationship between dementia and gut microbiome-associated metabolites: a cross-sectional study in Japan

**DOI:** 10.1038/s41598-020-65196-6

**Published:** 2020-05-18

**Authors:** Naoki Saji, Kenta Murotani, Takayoshi Hisada, Tadao Kunihiro, Tsuyoshi Tsuduki, Taiki Sugimoto, Ai Kimura, Shumpei Niida, Kenji Toba, Takashi Sakurai

**Affiliations:** 1 Center for Comprehensive Care and Research on Memory Disorders, National Center for Geriatrics and Gerontology, Aichi, Japan; 2 Biostatistics Center, Graduate School of Medicine, Kurume University, Fukuoka, Japan; 3 TechnoSuruga Laboratory Co., Ltd, Shizuoka, Japan; 4 Laboratory of Food and Biomolecular Science, Department of Bioscience and Biotechnology for Future Bioindustries, Graduate School of Agricultural Science, Tohoku University, Miyagi, Japan; 5 Medical Genome Center, National Center for Geriatrics and Gerontology, Aichi, Japan; 6 Department of Cognition and Behavioural Science, Nagoya University Graduate School of Medicine, Aichi, Japan

**Keywords:** Microbiology, Dementia, Risk factors

## Abstract

Dysregulation of the gut microbiome is associated with dementia. However, the relationship between microbiome-associated metabolites and dementia has yet to be identified. Outpatients visiting a memory clinic in Japan enrolled in this cross-sectional study; 107 subjects were eligible for the study, 25 of which had dementia. We collected demographics, activities of daily living, risk factors, cognitive function, and brain imaging data. The gut microbiome was assessed using terminal restriction fragment length polymorphism analysis. Concentrations of faecal metabolite were measured. We used multivariable logistic regression analyses to identify whether metabolites were independently related to dementia. The concentrations of metabolites were significantly different between subjects with and those without dementia. Every 1 standard deviation increment in faecal ammonia concentration was associated with around a 1.6-fold risk for the presence of dementia. A higher faecal lactic acid concentration was related to a lower risk of dementia, by around 60%. A combination of higher faecal ammonia and lactic acid concentrations was indicative of the presence of dementia, and had a similar predictive value as traditional biomarkers of dementia. Thus, faecal ammonia and lactic acid are related to dementia, independently of the other risk factors for dementia and dysregulation of the gut microbiome.

## Introduction

An estimated 47 million people worldwide were living with dementia in 2015, and this number is expected to triple by 2050^1^. The number of patients with dementia in Japan is also increasing, and an estimated 20% of the over-65 Japanese population will have dementia in the mid-2020s^2^. Therefore, a comprehensive strategy for dementia research has been introduced in Japan^3^. This strategy aims to promote social awareness of dementia, employ multifactorial assessments of clinical data, and determine the unknown risk of dementia, which could alleviate the increasing burden of dementia in Japan’s ageing population.

Recent research has identified novel associations between the gut microbiome and dementia^4–7^. Such work has suggested that the gut microbiome contributes to amyloid deposition, a strong risk factor for Alzheimer’s Disease (AD)^6^, and modulates host brain function via a microbiome–gut–brain axis^5,7^. Furthermore, dysregulation of the gut microbiome increases the risk of dementia independent of the other traditional risk factors^4^. The presence of bacterial products, such as microbiome-associated metabolites, in the systemic circulation may also increase inflammation which could lead to dementia^8^.

Nevertheless, it is not yet known how the gut microbiome and microbiome-associated metabolites affect cognitive function, and there have been conflicting findings regarding this association between the gut microbiome and dementia. For example, both decreased^4,5^ and increased^6^ proportions of *Bacteroides* have been reported in patients with dementia. Furthermore, while some work has indicated the effects of *Bacteroides* could increase the risk of dementia^6^, other work has suggested that *Bacteroides* could reduce the risk of cognitive decline^5^. In detail, Vogt *et al*.^6^ suggested that lipopolysaccharide, which is a component of the outer leaflet of the outer membrane of bacteria such as *Bacteroidetes*, potentiates systemic inflammation, amyloid fibrillogenesis, and results in amyloid deposition. Conversely, Alkasir *et al*.^5^ have proposed that *Bacteroidetes* regulate endothelial function and reduce inflammation. The protective effects of *Lactobacillus* have also been reported to have a protective effect against dementia via system activity and neurotransmitter release^5^.

In a recent clinical study, we investigated the association between gut microbiome composition, activities of daily living (ADL), and cognitive function^4^. In that study, we demonstrated that the gut microbiome is cross-sectionally associated with the presence of dementia^4^. We also found that the presence of both dementia and cardiovascular risk factors are associated with advanced dysregulation of the gut microbiome^9^. However, the mechanism underlying the relationship between the gut microbiome and cognitive function has not yet been clarified. In the present study, we assessed metabolite concentrations of the gut microbiome and analysed their independent association with dementia. We hypothesised that higher concentrations of faecal metabolites of the gut microbiome are associated with the presence of dementia, independent of the gut microbiome and other traditional risk factors.

## Results

### Patient characteristics

We analysed 128 subjects in the Gimlet study. Of these, 21 were excluded due to insufficient faecal samples. Therefore, data from a total of 107 eligible patients were analysed (female: 58.9%; mean age: 74.4 ± 7.9 years; mean MMSE score = 24). The group without dementia included 82 patients (76.6%) and the dementia group included 25 patients (23.4%) (Table 1).

**Table 1:** Patient characteristics.

	Total	Dementia group	No-dementia group	*P*
	(*n* = 107)	(*n* = 25)	(*n* = 82)	
***Demographics***				
Age, years	76, 69–81	75, 73–81	76, 68–80.3	0.479
Female sex, *n* (%)*	63 (58.9)	20 (80)	43 (52.4)	0.020
Education, years	12, 9–13	12, 10–13	12, 9–13	0.658
Body mass index, kg/m^2^	22.6, 20.5–24.4	22.8, 20.2–25.0	22.6, 20.6–24.1	0.669
***Risk factors***				
Hypertension, *n* (%)	66 (61.7)	19 (76.0)	47 (57.3)	0.106
Diabetes mellitus, *n* (%)	16 (15.0)	6 (24.0)	10 (12.2)	0.198
Dyslipidaemia, *n* (%)	51 (47.7)	15 (60.0)	36 (43.9)	0.177
CKD, *n* (%)	35 (32.7)	11 (44.0)	24 (29.3)	0.224
IHD, *n* (%)	12 (11.2)	5 (20.0)	7 (8.5)	0.146
History of stroke, *n* (%)	10 (9.3)	4 (16.0)	6 (7.3)	0.238
Smoking habit, *n* (%)	27 (25.2)	3 (12.0)	24 (29.3)	0.115
Alcohol consumption, *n* (%)	42 (39.3)	9 (36.0)	33 (40.2)	0.817
ApoE ε4 carrier, *n* (%)*	33 (30.8)	15 (60.0)	18 (22.0)	<0.001
***Comprehensive geriatric assessment***				
Barthel index	100	100, 95–100	100	0.065
IADL impairment, *n* (%)*	48 (44.9)	19 (76.0)	29 (35.4)	<0.001
DBDS*	8, 4–14	12, 6.5–16.5	7.5, 3.8–14	0.038
GDS	2, 1–5	2, 1–4.5	2.5, 1–5	0.520
Vitality index	10, 9–10	9, 8–10	10, 9–10	0.084
ZBI*	11, 3–22	18, 8.5–27.5	8.5, 3–18.3	0.010
MNA-SF	12, 11–13	12, 11–13	13, 11–13	0.053
***Cognitive function***				
MMSE score*	24, 21–28	19, 16–19	27, 23–29	<0.001
CDR-GB*				<0.001
0, *n* (%)	21 (19.6)	0 (0)	21 (25.6)	
0.5, *n* (%)	72 (67.3)	11 (44.0)	61 (74.4)	
1, *n* (%)	13 (12.1)	13 (52.0)	0	
2, *n* (%)	0 (0)	0 (0)	0	
3, *n* (%)	1 (1.0)	1 (4.0)	0	
CDR-SB*	2.0, 0.5–3.5	4.5, 3–5	1, 0.5–2.5	<0.001
***MRI findings***				
SLI, *n* (%)*	11 (10.3)	8 (32.0)	3 (3.7)	<0.001
WMH, *n* (%)	29 (27.1)	7 (28.0)	22 (26.8)	1.000
CMBs, *n* (%)	23 (21.5)	9 (36.0)	14 (17.1)	0.055
CSS, *n* (%)	7 (6.5)	3 (12.0)	4 (4.9)	0.350
VSRAD*	1.01, 0.65–2.03	2.07, 1.19–2.48	0.85, 0.56–1.42	<0.001
***Blood flow reduction seen in SPECT images***				
Posterior cingulate gyrus and/or precuneus, *n* (%)	72 (71.3)	19 (82.6)	53 (68.0)	0.201

Data are represented as the mean ± standard deviation or median (interquartile range) or number of patients (%).

Wilcoxon signed-rank and χ^2^ tests were used.

Asterisks indicate statistical significance (*P* < 0.05).

Abbreviations: CKD, chronic kidney disease; IHD, ischemic heart disease; ApoE, apolipoprotein E; IADL, instrumental activities of daily living; DBDS, Dementia Behaviour Disturbance Scale; GDS, Geriatric Depression Scale; ZBI, Zarit Caregiver Burden Interview; MNA-SF, Mini-Nutritional Assessment-Short Form; MMSE, Mini-Mental State Examination; CDR-GB, Clinical Dementia Rating Global Score; CDR-SB, Clinical Dementia Rating-Sum of Boxes; SLI, silent lacunar infarct; WMH, white matter hyperintensity; CMB, cerebral microbleeds; CSS, cortical superficial siderosis; VSRAD, voxel-based specific regional analysis system for Alzheimer’s disease; SPECT, single photon emission computed tomography. Enterotype I: Bacteroides >30%, Enterotype II: Prevotella >15%, Enterotype III: others.

*The number of patients who had identifiable data of each metabolite were as follows: Ammonia (*n* = 109), succinic acid (*n* = 52), lactic acid (*n* = 38), formic acid (*n* = 13), acetic acid (*n* = 96), propionic acid (*n* = 73), iso-butyric acid (*n* = 73), n-butyric acid (*n* = 82), iso-valeric acid (*n* = 58), n-valeric acid (*n* = 88), phenol (*n* = 89), P-cresol (*n* = 91), 4-ethylphenol (*n* = 66), indol (*n* = 96), and skatole (*n* = 51). The remaining patients had undetectable (extremely low) metabolites; these undetectable data were assigned values using the maximum likelihood estimation procedure^54^, which is half of the prescribed determination limit.

### Dementia vs. no-dementia group

Compared with the no-dementia group, the dementia group included significantly more women (Dementia *vs*. no-dementia: female sex, 80.0% *vs*. 52.4%, *P* = 0.02) and had impaired instrumental ADL (76.0% vs. 35.4%, *P* < 0.001), and a significantly lower cognitive function (median MMSE score, 19 *vs*. 27, *P* < 0.001; median CDR-SB score, 4.5 *vs*. 1.0, *P* < 0.001). Further, patients with dementia scored significantly lower on the Lawton and Brody scale, the Mini-Nutritional Assessment-Short Form (MNA-SF), Alzheimer’s Disease Assessment Scale–cognitive subscale (ADAS-cog), Frontal Assessment Battery (FAB), Raven’s Coloured Progressive Matrices, and Logical Memory subtests I and II of the Wechsler Memory Scale-Revised. Cerebral small vessel diseases such as silent lacunar infarcts (SLIs) and cerebral microbleeds (CMBs) and high VSRAD scores (the voxel-based specific regional analysis system for Alzheimer’s disease: VSRAD) were frequent on MRI scans of subjects with dementia (Table 1, S1). The dementia group had fewer enterotype I microbes (a lower prevalence of *Bacteroides*) and more enterotype III microbes (a higher prevalence of other bacteria) than the no-dementia group (20.0% *vs*. 46.3%, 80.0% *vs*. 47.6%, *P* = 0.014, respectively, Table 2). Although constipation is a microbiome-related factor^10^, there was no significant relationship between constipation and cognitive function. There were also no significant differences in metabolites between patients with and without constipation (Table S2).

**Table 2:** Gut microbiome of the patients.

	Total	Demented	Non-Demented	*P*
	(*n* = 107)	(*n* = 25)	(*n* = 82)	
***Gut microbiota***				
Enterotype*				0.001
Enterotype I	43 (40.2)	5 (20.0)	38 (46.3)	0.014
Enterotype II	5 (4.7)	0 (0)	5 (6.1)	
Enterotype III	59 (55.1)	20 (80.0)	39 (47.6)	
F/B ratio	1.50, 0.81–2.31	1.66, 1.09–3.00	1.33, 0.72–2.17	0.059
***Metabolite*** *				
Ammonia, mg/g*	0.69, 0.46–1.01	0.83, 0.66–1.30	0.65, 0.44–0.95	0.026
Succinic acid, mg/g	0.03, 0.03–0.41	0.03, 0.03–0.09	0.03, 0.03–0.77	0.581
Lactic acid, mg/g	0.03, 0.03–0.41	0.03, 0.03–0.07	0.03, 0.03–8.94	0.235
Formic acid, mg/g*	0.05, 0.05–0.05	0.05, 0.05–0.05	0.05, 0.05–0.05	0.044
Acetic acid, mg/g	3.63, 1.47–7.64	3.90, 1.35–7.81	3.61, 1.45–7.75	0.868
Propionic acid, mg/g	0.83, 0.03–2.01	0.89, 0.03–2.63	0.77, 0.03–1.69	0.417
Iso-butyric acid*, mg/g	0.11, 0.03–0.22	0.20, 0.08–0.26	0.08, 0.03–0.19	0.023
n-butyric acid, mg/g	0.30, 0.03–0.86	0.33, 0.16–1.49	0.26, 0.03–0.81	0.094
Iso-valeric acid*, mg/g	0.13, 0.03–0.34	0.33, 0.03–0.50	0.03, 0.03–0.26	0.008
n-valeric acid, mg/g	0.43, 0.12–2.65	0.36, 0.12–0.67	0.43, 0.12–2.78	0.385
Phenol, μg/g*	1.14, 0.60–2.11	1.97, 0.79–2.99	1.05, 0.47–1.94	0.029
P-cresol, μg/g*	4.21, 0.15–118.07	57.5, 2.38–160.9	0.29, 0.13–79.11	0.014
4-Ethylphenoll, μg/g	0.36, 0.001–1.01	0.56, 0.001–0.92	0.36, 0.001–1.08	0.849
Indolel, μg/g	6.04, 0.24–30.43	13.5, 0.72–38.7	5.0, 0.19–26.58	0.063
Skatolel, μg/g	0.001, 0.001–4.14	0.001, 0.001–10.18	0.001, 0.001–3.39	0.861

Wilcoxon signed-rank and χ^2^ tests were used.

The asterisks indicate statistical significance (*P* < 0.05).

Abbreviations: F/B ratio, Firmicutes/Bacteroidetes ratio. Enterotype I: Bacteroides > 30%; Enterotype II: Prevotella > 15%; enterotype III: others.

### Metabolites

**Figure 1:**
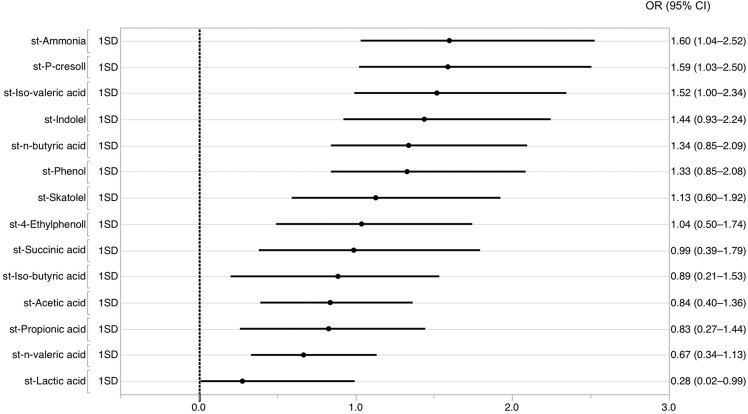
Univariable logistic regression analysis of the relationship between standardised metabolite values and the presence of dementia. Dots represent the ORs and lines represent the 95% CI for the presence of dementia.

The concentrations of some metabolites in faeces such as ammonia, phenol, and p-cresol were significantly higher in the dementia group compared with the no-dementia group (median ammonia: 0.83 *vs*. 0.65 mg/g, *P* = 0.026; median phenol: 1.97 *vs*. 1.05 μg/g, *P* = 0.029; median p-cresol: 57.5 *vs*. 0.29 μg/g, *P* = 0014, respectively, Table 2). Univariable analyses to identify the metabolites that most significantly contributed to the presence of dementia showed that the highest OR was of faecal ammonia concentration (OR = 1.6, 95% CI 1.0–2.5, *P* = 0.033), the lowest OR was of faecal lactic acid concentration (OR = 0.3, 95% CI 0.02–1.0, *P* = 0.048), and OR range of the other metabolites was 0.7–1.6 (Fig. 1, Table S3). Because medication is a microbiome metabolite-related factor^11^, we conducted bivariable analyses between each microbiome metabolite and a range of medications. The use of some medications (anti-thrombotic drug, statin, anti-hyperglycaemic drug, and aperient) was associated with faecal metabolite levels such as lactic acid, acetic acid, iso-valeric acid, n-butyric acid, phenol, 4-Ethylphenoll, and indolel (Table S4).

### Multivariable analysis

**Figure 2:**
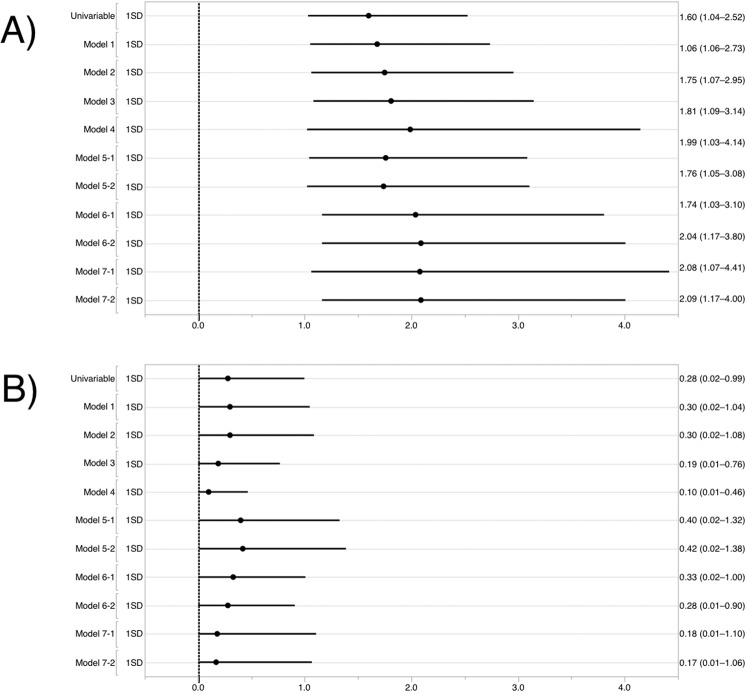
Logistic regression analysis of standardised (**A**) ammonia concentration and (**B**) lactic acid concentration in faecal samples and their relationship with dementia presence. Dots represent the ORs and lines represent the 95% CI for the presence of dementia.

Multivariable logistic regression analysis revealed that ammonia concentration was associated with the presence of dementia, independent of age, sex, education years, ApoE ε4, enterotypes (either enterotype I or III), and risk factors (Table 3, S5). The overall ORs of ammonia after adjustment were at least 1.6 (Fig. 2A, Table S6). Multivariable logistic regression analysis revealed that lactic acid concentration was associated with the presence of dementia, independent of age, sex, education years, ApoE ε4, risk factors, and brain MRI and/or SPECT abnormalities (OR = 0.1, 95% CI 0.01–0.5, *P* = 0.001, Table S7). Lactic acid concentration had a tendency to exhibit an inverse association with the presence of dementia (Table 3, S5). The overall ORs of lactic acid after adjustment were at most 0.4 (Fig. 2B, Table S7).

**Table 3:** Multivariable logistic regression analysis of standardised ammonia and lactic acid concentrations in faecal samples for the presence of dementia.

	OR	95% CI	*P*
**Model 1**			
st-Ammonia*	1.7	1.1–2.8	0.023
st-Lactic acid	0.3	0.02–1.0	0.051
**Model 2**			
st-Ammonia*	1.8	1.1–3.0	0.021
st-Lactic acid	0.3	0.02–1.0	0.058
**Model 3**			
st-Ammonia*	1.8	1.1–3.1	0.023
st-Lactic acid	0.4	0.02–1.2	0.125
Enterotype I	0.5	0.1–1.8	0.258
F/B ratio	1.2	1.0–1.5	0.124
**Model 4**			
st-Ammonia*	1.8	1.1–3.2	0.031
st-Lactic acid	0.4	0.02–1.3	0.154
Enterotype III	3.6	0.9–16.0	0.073
F/B ratio	1.1	0.9–1.4	0.270

The dependent variable was the prevalence of dementia.

The asterisk indicates statistical significance (*P* < 0.05).

Model 1: adjusted for age, sex, education years.

Model 2: adjusted for model 1 and the presence of ApoE ε4.

Model 3: adjusted for model 2, enterotype I, and the F/B ratio.

Model 4: adjusted for model 2, enterotype III, and the F/B ratio.

Abbreviations: OR, odds ratio; CI, confidence interval. F/B ratio, Firmicutes/Bacteroidetes ratio.

These statistical models demonstrated that the combination of a higher level of ammonia and a lower level of lactic acid concentration resulted in the highest AUC score (sensitivity, specificity, and AUC: a combination of ammonia and lactic acid; 62%, 76%, 0.69; ammonia alone; 50%, 80%, 0.65; lactic acid alone; 32%, 88%, 0.57, respectively) and showed similar or slightly lower AUC scores compared with traditional biomarkers of dementia, such as ApoE ε4 and VSRAD scores (sensitivity, specificity, and AUC: ApoE ε4; 78%, 60%, 0.69; VSRAD score; 86%, 63%, 0.80, respectively). All multivariable models converged.

### Graphical modelling

**Figure 3:**
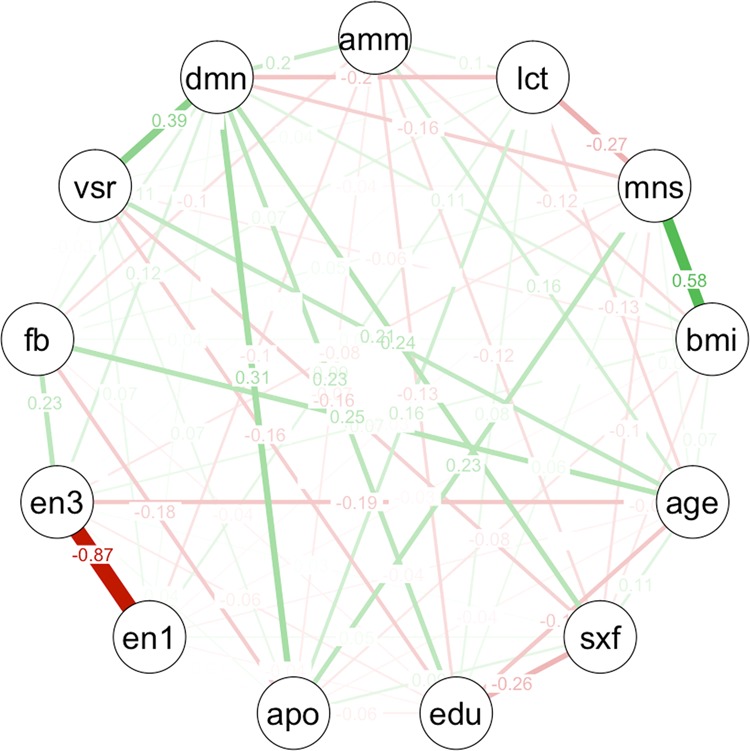
Graphical modelling of all variables. Line thickness is proportional to the number of patients that contributed to the comparison. Green lines indicate a positive relationship, red lines indicate a negative relationship. Abbreviations: amm, standardised faecal ammonia concentration; lct, standardised faecal lactic acid concentration; mns, Mini-Nutritional Assessment-Short Form; bmi, body mass index; sxf, female sex; edu, education year; apo, ApoE ε4 carrier; en1, enterotype I; en3, enterotype III; fb, F/B ratio; vsr, VSRAD score; dmn, the presence of dementia.

The graphical modelling showed that the presence of dementia was more likely to be associated with a higher VSRAD score, the presence of ApoE ε4, female sex, less years of education, and metabolite concentrations. Metabolites such as a higher concentration of ammonia and a lower concentration of lactic acid showed strong associations with dementia compared with enterotypes (line thickness between each clinical feature and dementia; ammonia, lactic acid, enterotype I, and enterotype III: 0.2, –0.2, –0.1, and 0.12, respectively). Furthermore, we found a stronger association between enterotypes I and III compared with the association between enterotypes and dementia (line thickness between enterotype I and enterotype III, enterotype I and dementia, enterotype III and dementia: –0.87, –0.1, and 0.12, respectively, Fig. 3).

## Discussion

The main finding of this sub-analysis study was a relationship between gut microbiome-associated metabolites and dementia. More specifically, every 1 SD increment in faecal ammonia concentration was associated with around a 1.6-fold increased risk for the presence of dementia. We also found that every 1 SD decrement in faecal lactic acid concentration tended to be associated with a reduced risk of dementia. A combination of faecal ammonia and lactic acid concentration was indicative of the presence of dementia and had the same or slightly lower predictive accuracy as traditional biomarkers of dementia, including ApoE ε4 and VSRAD scores.

The independent relationship between the faecal metabolite concentration and dementia shown in our study is novel. Existing literature indicates that gut microbial metabolites provide a functional readout of microbial activity and can be used as an intermediate phenotype mediating host-microbiome interactions^12^. Furthermore, metabolites such as phenol, p-cresol, indole, and ammonia are typically considered to be potentially harmful^13^. The levels of these metabolites in our study were higher in demented patients than in non-demented patients. Our finding is in line with previous studies^12,13^. To the best of our knowledge, this is the first clinical study to demonstrate the association of gut microbiome metabolites – ammonia and lactic acid concentration – with cognitive function in the elderly. We found that faecal ammonia was positively related to the presence of dementia. A high blood ammonia concentration is known to be a risk factor for cognitive impairment^14^ and Alzheimer’s disease^15^, and our findings are in line with these reports. Higher faecal ammonia concentrations may be related to higher blood ammonia concentrations^13^. Furthermore, faecal ammonia concentrations in children with autism spectrum disorders are higher than those in control children^16^. Ammonia alters nucleic acid synthesis, changes morphology and intermediary metabolism, and reduces the life span of intestinal cells^17^. These previous findings also support our findings in the current study. Short-chain fatty acids (SCFAs) were also associated with dementia in the present study. SCFAs produced by gut microbes can regulate the biosynthesis of neurotransmitters, such as serotonin^18^ and leptin^19^. Although we did not identify any neurotransmitters related to SCFAs that might regulate host cognitive function in our study, dysbiosis may affect the biosynthesis of neurotransmitters, affecting brain function. Previous findings of associations between the gut microbiome and depression^20^ or attention deficit hyperactivity disorder^21^ support this speculation.

Inversely, our multivariable analysis revealed that the OR of lactic acid for dementia was at most 0.4, although there was not a significant independent association with dementia. This means that lactic acid may have a protective effect against dementia. Indeed, several previous studies have reported *Lactobacillus*^22^ and/or *Bifidobacterium* to have protective effects against cognitive deterioration^23,24^. The relationship between lactic acid and dementia may be due to the direct activation of lactic acid-producing bacterium such as *Lactobacillus* or *Bifidobacterium*, or the secondary benefit of consuming food and drink containing a lactic acid fermentation product. We did not identify the origin of the faecal lactic acid (endogenous or exogenous) and this should be addressed in future studies.

The mechanism underlying the relationship between faecal metabolite concentration and dementia has yet to be clarified. However, we found an independent association between the two on the basis of our systematic assessment of cognitive function using a comprehensive geriatric assessment, various faecal metabolites, and potential biomarkers for dementia. The graphical modelling also showed such relationship. This relationship might be caused by functional disorders of the neuro-inflammatory system^25^, microvascular inflammation^8^, or remote effects driven by various metabolites^26^. Specifically, previous studies have speculated on the potential mechanisms connecting faecal metabolites and dementia. Gut microbiome disorder promotes the production of toxic metabolites and inflammation-mediated cytokines, and a reduction in beneficial substances such as anti-inflammatory factors. There are functional disorders of the gut epithelial barrier, with concomitant activation of the immune system, as well as the dysregulation of enteric neurons and glia^27^. These events subsequently lead to blood–brain barrier dysfunction, triggering neuro-inflammatory reactions and apoptotic neuronal and glial cell death, particularly in the hippocampus and cerebral cortex, which underlie the development of dementia^27^. Recent studies have also reported that microbiome metabolites, such as SCFA and trimethylamine/trimethylamine N-oxide, contribute to life-threatening diseases such as heart failure and atherosclerosis^8^. Our comprehensive assessment can provide detailed evidence to fill the knowledge gap regarding the mechanism between dementia and the gut microbiome. Furthermore, analysing metabolites of the gut microbiome longitudinally could tell us more about the relationship between gut microbiome dysfunction and dementia, and could reveal the unidentified mechanism for the onset of dementia.

Our study has limitations. The causal relationship between metabolites and dementia could not be tested because this is a cross-sectional study. We are in the process of conducting a longitudinal assessment in the schema of the Gimlet study and this assessment will clarify the causal relationship. The relatively small number of subjects may mean that there was a low statistical power. There might be a selection bias because of a single hospital-based cohort. There might be possible differences in collection and storage of faecal samples among study subjects. High-throughput DNA sequencing technology would be useful to identify the specific genera or species of microbes compared with the terminal restriction fragment length polymorphism (T-RFLP) method^4^. The types of gut microbiome in an individual can be affected by age^28^, diet^29^, and medication^6,30,31^. We have recently assessed patients’ diet patterns and will analyse the associations between cognitive function, gut microbiome, and diet patterns in a forthcoming sub-analysis. Regarding medication, although the use of some drugs was associated with metabolites in our bivariable analyses, it is difficult to find clinically consistent interpretations of these associations. The use of any anti-thrombotic drugs may be associated with underlying cardiovascular or cerebrovascular diseases. In addition, the use of aperient may be associated with constipation. Furthermore, the use of gastric acid-suppressing drugs might affect the gut microbiome, because a previous study has reported that proton pump inhibitors reduce gastric acid secretion and may modulate gut microbiota composition^30^. Thus, we did not include medication in the multivariable analyses because the effects of medication on cognitive function are more complex than our study allows. Frailty^32^ may also affect the gut microbiome, and should be investigated in further studies. Amyloid β precursor protein may also be important to assess, because a high serum concentration of amyloid β precursor protein is indicative of inflammatory endothelial dysfunction and increased the risk of cognitive impairment^33^. Furthermore, subtypes of dementia, such as Alzheimer’s disease and frontotemporal lobar degeneration, were not considered in the current sub-analysis, because our sample size was relatively small; subdividing patients by dementia subtypes would lead to a small number of subjects in each group and a resulting statistical underestimation. The main aim of the present study was to determine the association between the gut microbiome and dementia, which was defined by simple categorisation on the basis of MMSE and CDR scores. However, the absence of any analysis of dementia subtypes limits the utility of our findings, and will be corrected in future studies.

Although this sub-analysis study included a small number of subjects and we did not show a robust and statistically significant relationship regarding the association between lactic acid and dementia in the multivariable analyses, our findings, at least regarding faecal ammonia, support the idea that there is a relationship between the gut microbiome and dementia. Further studies are warranted to clarify the mechanism underlying such relationship.

## Conclusions

We have shown a strong relationship between gut microbiome-associated metabolites such as ammonia and dementia, independent of the traditional risk factors and the gut microbiome. Furthermore, faecal lactic acid concentration may be inversely associated with the presence of dementia.

## Methods

### Study design

This study was a sub-analysis of data from the gerontological investigation of microbiome: a longitudinal estimation study (the Gimlet study) at the National Center for Geriatrics and Gerontology (NCGG)^4^. The present sub-analysis study investigated the cross-sectional association between metabolites of the gut microbiome and dementia. The study complied with the tenets of the Declaration of Helsinki and was approved by the Institutional Review Board at the NCGG (No. 1191). Informed consent was obtained from all patients and their families before participating in this study. The study is registered with the UMIN Clinical Trials Registry (UMIN000031851). Detailed information regarding the Gimlet study and the study methods is provided in the supplementary file and the previous report^4^.

### Subjects

Between March 2016 and March 2017, we enrolled consecutive patients visiting the memory clinic at the NCGG who agreed to undergo medical assessment of their cognitive function and faecal examination. Patients were eligible for participation in the Gimlet study if they met the following criteria: (1) able to undergo brain magnetic resonance imaging (MRI); (2) provided written informed consent; (3) provided informed consent for the NCGG Biobank to store their clinical data, blood, and faecal samples; and (4) were accompanied by a study partner who could assess the daily condition of the patient (patient’s family or a primary caregiver). The exclusion criteria were as follows: (1) the MRI scan could not be evaluated because of movement-related artefacts; (2) local lesions, such as cerebral infarction, that were detected by MRI before enrolment, which could significantly affect cognitive functioning; (3) a history of a major psychological disorder or current serious or unstable alcohol or drug abuse; (4) ≤6 years of education, because a low level of education is a robust risk factor for dementia; (5) a history of cancer of the digestive tract; (6) brain tumour, encephalitis/meningitis, normal pressure hydrocephalus, subdural hematoma, or lower cognitive function due to head injury; and/or (7) were unable to provide sufficient faecal samples for metabolite analysis.

### Baseline assessment

All participants underwent a comprehensive geriatric assessment^34^ that measured the following: (1) demographic characteristics; (2) potential risk factors for dementia, such as hypertension, dyslipidaemia, diabetes mellitus, ischemic heart disease, chronic kidney disease, a smoking habit, a history of stroke, and alcohol consumption; (3) the Barthel Index to assess basic ADL^35^, and the Lawton and Brody scale to assess instrumental ADL^36^; (4) global cognitive function, using the Mini-Mental State Examination (MMSE)^37^ and Clinical Dementia Rating (CDR) scale^38^; (5) neuropsychological tests, including the Alzheimer’s Disease Assessment Scale–cognitive subscale^39^, Frontal Assessment Battery^40^, Raven’s Coloured Progressive Matrices^41^, and Logical Memory subtests I and II of the Wechsler Memory Scale-Revised^42^; (6) laboratory variables, including ApoE ε4 as a risk factor for AD; (7) ankle brachial index and pulse wave velocity as indicators of arteriosclerosis^43^, and the ‘impact’ of pulse^44^; (8) brain imaging such as MRI and single photon emission-computed tomography (SPECT); (9) assessment of other clinical features such as frailty^45^ and subjective hearing loss; (10) social and lifestyle factors, such as nutritional status, which was measured using the MNA-SF^46^, and constipation, which was defined as a bowel movement once in 3 days and/or the taking of anti-constipation drugs (aperients); and (11) assessment of current medication (anti-dementia drugs, anti-hypertensive drugs, statins, proton pump inhibitors/H2 blockers, anti-thrombotic drugs, anti-hyperglycaemic drugs, and aperients). The clinical samples and data were provided by the NCGG Biobank, which collects clinical data for research.

### Classification of cognitive function

We divided patients into the two following categories according to our previous report:^4^ (1) the group without dementia (MMSE score ≥20 and a CDR score <1) and the group with dementia (MMSE score <20 and/or a CDR score ≥1). A CDR score of 0.5 indicates the presence of mild cognitive impairment (MCI) and possibly very mild dementia, which means that the patient has a higher risk of dementia^47^. Normal cognition was defined as MMSE ≥ 20 and CDR = 0.

### Brain imaging

Patients underwent a 1.5 T MRI brain scan (Philips Ingenia, Eindhoven, the Netherlands). MRI scans obtained included diffusion-weighted imaging, fluid-attenuated inversion recovery imaging, T2-weighted imaging, T2^*^-weighted gradient echo imaging, 3D T1-weighted sagittal and axial coronal views, and 3D time-of-flight MR angiography scans. The presence and components of cerebral small vessel disease were categorised using the standards for reporting vascular changes on neuroimaging^48^, including SLIs, white matter hypersensitivity (WMH), CMBs, and cortical superficial siderosis (CSS). We used VSRAD advance software (Eisai Co., Ltd., Tokyo, Japan) to quantify cortical and hippocampal atrophy using a standardised z-score. An increased VSRAD score suggests the potential presence of AD because it is indicative of hippocampal atrophy, one of the characteristics of the AD brain^49^. Patients also underwent N-isopropyl-p-[^123^I]-iodoamphetamine-SPECT to assess the presence or absence of a reduction in blood flow in the posterior cingulate gyrus and/or praecuneus as a surrogate marker of Alzheimer’s disease^50^.

### Gut microbiome

Faecal samples were collected at home and were frozen and preserved at −81 °C at the NCGG Biobank. Patients were consuming their usual diet at the time of sampling. After all samples had been collected, the gut microbiome was analysed using T-RFLP analysis by the TechnoSuruga Laboratory (Shizuoka, Japan)^51^. T-RFLP analysis is one of the most well-established and reliable 16 S ribosomal RNA-based methods, which has a high throughput and reproducibility^51^. First, T-RFLP was used to classify gut microbes into the following 10 groups: *Prevotella, Bacteroides*, Lactobacillales*, Bifidobacterium, Clostridium* cluster IV*, Clostridium* subcluster XIVa*, Clostridium* cluster IX*, Clostridium* cluster XI*, Clostridium* cluster XVIII, and ‘others’. Second, we stratified the gut microbiome into the three following enterotypes: enterotype I included *Bacteroides* at >30%, enterotype II included *Prevotella* at >15%, and enterotype III included the remaining bacteria, and this classification was made according to the Human Faecal Microbiome T-RFLP profile^52,53^. Third, we assessed the Firmicutes/Bacteroidetes (F/B) ratio^53^. The phylum Firmicutes includes the Lactobacillales and the *Clostridium* clusters, and the phylum Bacteroidetes includes *Bacteroides* and *Prevotella*.

### Analysis of metabolites in faeces

We measured faecal levels of organic acids, SCFAs, ammonium ion, indoles, phenol, skatole, and p-cresol to determine metabolite levels. We measured organic acids and SCFAs such as acetic acid, propionic acid, butyric acid, iso-butyric acid, succinic acid, lactic acid, formic acid, valeric acid, and iso-valeric acid using high-performance liquid chromatography (Prominence, Shimadzu, Kyoto, Japan) with a detector (CDD-10A, Shimadzu, Kyoto, Japan), two tandemly-arranged columns (Shim-pack SCR-102(H), 300 mm × 8 mm ID, Shimadzu, Kyoto, Japan), and a guard column (Shim-pack SCR-102(H), 50 mm × 6 mm ID, Shimadzu, Kyoto, Japan). We measured ammonium ion concentration using an ion chromatography system (ICS-1000, DIONEX) with a column (IonPac CS12A, 4 mm × 250 mm, DIONEX) and a guard column (IonPac CG12A, 4 mm × 50 mm, DIONEX). We measured indoles, phenol, skatole, and p-cresol using gas chromatography/mass spectrometry (QP-2010, Shimadzu, Kyoto, Japan) and a capillary column (Inert cap WAX, 30 m × 0.25 mm × 0.25 µm, GL science, Japan). Detailed information regarding the analysis of metabolites in faeces is provided in the supplementary file.

### Statistical analysis

Continuous, ordinal, and categorical variables are expressed as the mean ± standard deviation (SD), median and interquartile range, and frequency or proportion (percentage), and were compared using the unpaired Student’s *t*-test, Wilcoxon rank-sum test, and χ^2^ test, respectively according to our previous study^4^. We compared clinical characteristics, composition of the gut microbiome, and metabolites between the no-dementia and dementia groups using the Wilcoxon rank-sum test and the χ^2^ test. Second, we standardised actual measurement values of metabolites and performed univariable logistic regression analyses to identify the metabolites that were associated with the presence of dementia. Third, multivariable logistic regression models were used to identify the metabolites independently associated with dementia using the standardised metabolite values. Backward stepwise multivariable logistic regression analyses were performed by adjusting for patient demographics, ApoE ε4, gut microbiome (enterotype and the F/B ratio), risk factors, brain MRI findings, and the blood flow reduction on SPECT images. Fourth, the sensitivity, specificity, and receiver operating characteristic (ROC) curve were calculated and the area under the ROC curve (AUC) was compared between the with and without dementia groups to evaluate the usefulness for predictive diagnosis of dementia. Finally, we used graphical modelling to visualise the mutual associations of items used in the multivariable logistic regression analyses, and to identify whether the gut microbiome and/or metabolites were strongly associated with the presence of dementia. Odds ratios are presented with 95% confidence intervals (CI). All comparisons were two-tailed, and *P* < 0.05 was considered to represent statistical significance. Data were analysed using the JMP 11.0 software package (SAS Institute Inc., Cary, NC) and the graphical modelling was generated using the R software (R Language and Environment for Statistical Computing, Vienna, Austria).

## Supplementary information

Supplementary file.

## Data Availability

The datasets have been deposited in the UMIN case data repository system (ID: UMIN000031851). The datasets used and/or analysed during the current study are available from the corresponding author on reasonable request: https://www.umin.ac.jp/icdr/index.html.
