# Supplementary material for: Relationship between dementia and gut microbiome-associated metabolites: a cross-sectional study in Japan
Source: Sci Rep. 2020 May 18;10:8088. doi: 10.1038/s41598-020-65196-6 (PMC7235213; doi:10.1038/s41598-020-65196-6)
Supplement: Supplementary file 1 — Supplementary file. [file 41598_2020_65196_MOESM1_ESM.docx]

**Supplementary file**

**Relationship between dementia and gut microbiome-associated metabolites: a cross-sectional study in Japan**

Naoki Saji^*1^, Kenta Murotani^2^, Takayoshi Hisada^3^, Tadao Kunihiro^3^, Tsuyoshi Tsuduki^4^, Taiki Sugimoto^1^, Ai Kimura^1^, Shumpei Niida^5^, Kenji Toba^1^, Takashi Sakurai^1,6^

^1^Center for Comprehensive Care and Research on Memory Disorders, National Center for Geriatrics and Gerontology, Aichi, Japan

^2^Biostatistics Center, Graduate School of Medicine, Kurume University, Fukuoka, Japan

^3^TechnoSuruga Laboratory Co., Ltd, Shizuoka, Japan

^4^Laboratory of Food and Biomolecular Science, Department of Bioscience and Biotechnology for Future Bioindustries, Graduate School of Agricultural Science, Tohoku University, Miyagi, Japan

^5^Medical Genome Center, National Center for Geriatrics and Gerontology, Aichi, Japan

^6^Department of Cognition and Behavioural Science, Nagoya University Graduate School of Medicine, Aichi, Japan

**Supplementary Methods**

***MRI***

Patients underwent 1.5T magnetic resonance imaging (MRI) of the brain (Philips Ingenia, Eindhoven, the Netherlands), including diffusion-weighted imaging, fluid-attenuated inversion recovery (FLAIR) imaging, T2^*^-weighted gradient echo imaging (T2WI), and 3D time-of-flight MR angiography. A recent small subcortical infarct (RSSI) was defined as an infarct of <15 mm in diameter, detected using diffusion-weighted imaging. A silent lacunar infarct was defined as a focal lesion of ≥3 mm in diameter that was hyperintense on T2WI and hypointense on FLAIR images. White matter hyperintensity was defined as an irregular periventricular hyperintensity (Fazekas grade ≥3) and/or early confluent or confluent separate deep hyperintense lesions (Fazekas grade ≥2) in the white matter on T2WI and FLAIR images. A cerebral microbleed was defined as a focal area of signal loss of <5 mm in the brain parenchyma on a T2^*^ scan. Cortical superficial siderosis was defined as a homogeneous hypointense curvilinear signal intensity (black) on a T2^*^ scan in the superficial layers of the cerebral cortex, within the subarachnoid space, or both.

***Risk factors***

Hypertension was defined by a systolic blood pressure of ≥140 mmHg or a diastolic blood pressure of ≥90 mmHg, and/or the use of anti-hypertensive drugs. Dyslipidaemia was defined by a serum low-density lipoprotein cholesterol concentration of ≥140 mg/dL, a serum high-density lipoprotein cholesterol concentration of <40 mg/dL, a serum triacylglycerol concentration of ≥150 mg/dL, and/or the use of statins. Diabetes mellitus was defined by a haemoglobin A1c (HbA1c) concentration of ≥6.5%, and/or the use of oral hypoglycaemic drugs or insulin, and/or a fasting serum glucose concentration of ≥69.9 mol/L (126 mg/dL). Ischaemic heart disease was defined by a history of physician-diagnosed angina pectoris and/or evidence of a prior myocardial infarction or coronary revascularisation procedure (percutaneous coronary intervention or coronary artery bypass surgery). Serum creatinine was measured and the estimated glomerular filtration rate (eGFR) was determined using the equation proposed by the Japanese Society of Nephrology, as follows: eGFR (mL/min/1.73 m^2^) = 194 × (serum creatinine [mg/dL])^−1.094^ × (age [years])^−0.287^ (× 0.739 if female). Chronic Kidney Disease (CKD) was defined by an eGFR of <60 mL/min/1.73 m^2^.

***Sample collection***

Patients or their family members used a faecal sampler to collect a faecal sample as soon as possible after the patient’s bowel movement, and the sample was placed in a specimen container. Patients collected faecal samples on the day of their hospital consultation, and the samples were presented to the clinical laboratory centre of the National Center for Geriatrics and Gerontology (NCGG; preferably within 4 h of bowel movement). Because one of the inclusion criteria for our study was that patients presented with study partners (family members), all of the demented patients were able to be supported if they needed support about excretion. Furthermore, when we received the faecal samples, we only accepted appropriate samples for the analysing company’s suggestion, while inadequate samples were disposed of and resubmission was requested. The samples were frozen and stored at −81°C in the NCGG Biobank. After all the samples had been collected, they were transported (frozen) to the TechnoSuruga Laboratory (Shizuoka, Japan).

***Analysis of metabolites in faeces***

*Organic acid*

To determine the presence of organic acid, 0.1 g of faeces was put in a 2.0-mL tube with zirconia beads and suspended in MilliQ water. Samples were heated at 85°C for 15 min, vortexed at 5 m/s for 45 s using FastPrep 24 (MP Biomedicals, CA, USA), and centrifuged at 15,350 × *g* for 10 min. The supernatant was filtrated using a 0.2-µm filter. Organic acids (acetic acid, propionic acid, butyric acid, iso-butyric acid, succinic acid, lactic acid, formic acid, valeric acid, and iso-valeric acid) in faeces were measured using high-performance liquid chromatography (Prominence, Shimadzu, Kyoto, Japan) with a detector (CDD-10A, Shimadzu, Kyoto, Japan), two tandemly-arranged columns (Shim-pack SCR-102(H), 300 mm × 8 mm ID, Shimadzu, Kyoto, Japan), and a guard column (Shim-pack SCR-102(H), 50 mm × 6 mm ID, Shimadzu, Kyoto, Japan). A 5 mM p-toluenesulfonic acid solution was used at the mobile phase and a mixture (5 mM p-toluenesulfonic acid, 100 µM EDTA, and 20 mM Bis-Tris) was used at the reaction solution. The flow rate and oven temperature were 0.8 mL/min and 45°C, respectively. The detector cell temperature was maintained at 48°C. The measurements were performed with an absolute calibration curve (range from 5 to 1,000 mg/L).

*Ammonia*

To measure ammonium ion concentration, 0.1 g of faeces was suspended in 0.9 mL MilliQ, heated at 85°C for 15 min, and filtered through a 0.20-µm filter. Samples were diluted 200-fold in MilliQ. Ammonium ion concentration was measured using an ion chromatography system (ICS-1000, DIONEX) with a column (IonPac CS12A, 4 mm × 250 mm, DIONEX) and a guard column (IonPac CG12A, 4 mm × 50 mm, DIONEX). In the mobile phase, a 10.7 mM sulfuric acid solution was used, and the flow rate was 1.0 mL/min. The detector temperature was maintained at 35°C. The measurements were performed with an absolute calibration curve (with a range of 0.025–2.0 mg/L).

*Indole, phenol, skatole, and p-cresol*

A total of 0.1 g of faeces was suspended in 2.5 mL phosphate buffer including 0.4 mg/L 4-isopropylphenol as an internal standard. The sample was heated at 85°C for 15 min, mixed with 2.5 mL acetonitrile and 1 g NaCl, shaken for 30 min, and centrifuged at 1,300 × *g* for 10 min. One millilitre of the supernatant was dehydrated and purified by a sodium sulfate drying cartridge (Bond Elut LRC, Agilent Technologies, USA), C18 cartridge (Smart SPE C18-30, AiSTI SCIENCE, Japan), and PSA cartridge (Smart SPE PSA-30, AiSTI SCIENCE, Japan), and placed into a vial.

Indoles and phenols were measured using gas chromatography/mass spectrometry (QP-2010, Shimadzu, Kyoto, Japan) and a capillary column (Inert cap WAX, 30 m × 0.25 mm × 0.25 µm, GL science, Japan). Helium was used as the carrier gas at 1.11 mL/min. The injector temperature and the interface temperature were kept at 240°C and 230°C, respectively. The oven temperature program was as follows: 70°C for 2 min; then rose by 20°C/min to 200°C, held for 3 min; rose by 10°C/min to 240°C; finally, 240°C, held for 16 min. One microliter of the extract was injected in the splitless mode. The mass spectrometer was operated in electron impact ionisation mode at 70 eV. The measurements were performed with an absolute calibration curve (range from 0.02 to 5.0 mg/L). Data acquisition was performed in selected ion monitoring (SIM) mode for quantification.

**Supplementary tables**

| **Table S1. Additional patient characteristics** | | | | |
| --- | --- | --- | --- | --- |
|  | Total |  |  |  |
|  |  | Dementia group | No-dementia group | *P* |
|  | (*n* = 107) | (*n* = 25) | (*n* = 82) |  |
| ***Cognitive function*** | | | | |
| ADAS-cog* | 9.0, 5.7–14 | 14.2, 12.6–16.6 | 7.6, 5.3–11.7 | <0.001 |
| RCPM* | 28, 24–32 | 26, 23–28 | 29, 24–32.5 | 0.016 |
| FAB* | 11.0, 9.0–13.0 | 9, 7–10.5 | 12, 10–14 | <0.001 |
| LM-WMSR I* | 8, 4–15 | 4, 1.5–5.5 | 10, 5.3–17.5 | <0.001 |
| LM-WMSR II* | 3, 0–8 | 0, 0–0 | 3.5, 0–10 | <0.001 |
| ***Arterial stiffness*** | | | | |
| Pulse wave velocity, m/s | 18.4, 15.9–21.8 | 19.3, 16.5–21.2 | 17.7, 15.8–22.0 | 0.425 |
| Ankle brachial index | 1.11, 1.05–1.15 | 1.12, 1.04–1.14 | 1.10, 1.07–1.15 | 0.692 |
| ***Medication*** | | | | |
| Anti-dementia drug, *n* (%) | 11 (10.3) | 5 (20.0) | 6 (7.3) | 0.124 |
| Anti-hyperglycaemic drug, *n* (%)* | 12 (11.3) | 6 (24.0) | 6 (7.4) | 0.033 |
| Anti-hypertensive drug, *n* (%) | 58 (55.2) | 15 (60.0) | 43 (53.8) | 0.649 |
| Statin, *n* (%) | 40 (38.1) | 12 (48.0) | 28 (35.0) | 0.250 |
| Anti-thrombotic drug, *n* (%) | 23 (21.9) | 8 (32.0) | 15 (18.8) | 0.175 |
| PPI/H2 blocker, *n* (%) | 27 (25.5) | 8 (32.0) | 19 (23.5) | 0.435 |
| Aperient, *n* (%) | 12 (11.3) | 3 (12.0) | 9 (11.1) | 1.000 |

Data are represented as the median (interquartile range) or number of patients (%). Wilcoxon signed-rank and χ^2^ tests were used.

Asterisks indicate statistical significance (*P* < 0.05).

Anti-dementia drugs: donepezil, rivastigmine, galantamine, and memantine.

Anti-hypertensive drugs: calcium channel blockers, angiotensin-converting-enzyme inhibitors, and angiotensin II receptor blockers.

Abbreviations: ADAS-cog, Alzheimer’s Disease Assessment Scale-Cognitive Subscale; RCPM, Raven’s Coloured Progressive Matrices; FAB, Frontal Assessment Battery; LM-WMSR, Logical Memory subtests I and II of the Wechsler Memory Scale-Revised; PPI, proton pump inhibitor.

| **Table S2. Patient characteristics regarding constipation** | | | | |
| --- | --- | --- | --- | --- |
|  | Total |  |  |  |
|  |  | Constipation group | No-constipation group | *P* |
|  | (*n* = 107) | (*n* = 25) | (*n* = 82) |  |
| ***Demographics*** | |  |  |  |
| Age, years | 76, 69–81 | 75, 68–79 | 76, 70–81 | 0.550 |
| Female sex, *n* (%) | 63 (58.9) | 13 (52.0) | 50 (61.0) | 0.489 |
| Education, years | 12, 9–13 | 12, 10–15 | 12, 9–12 | 0.149 |
| Body mass index, kg/m^2^ | 22.6, 20.5–24.4 | 23.7, 21.3–25.0 | 22.4, 20.3–24.2 | 0.170 |
| ***Risk factors*** |  |  |  |  |
| Hypertension, *n* (%) | 66 (61.7) | 16 (64.0) | 50 (61.0) | 0.819 |
| Diabetes mellitus, *n* (%) | 16 (15.0) | 7 (28.0) | 9 (11.0) | 0.053 |
| Dyslipidaemia, *n* (%) | 51 (47.7) | 12 (48.0) | 39 (47.6) | 1.000 |
| CKD, *n* (%) | 35 (32.7) | 7 (28.0) | 28 (34.2) | 0.633 |
| IHD, *n* (%) | 12 (11.2) | 5 (20.0) | 7 (8.5) | 0.146 |
| History of stroke, *n* (%) | 10 (9.3) | 3 (12.0) | 7 (8.5) | 0.696 |
| Smoking habit, *n* (%) | 27 (25.2) | 5 (20.0) | 22 (26.8) | 0.604 |
| Alcohol consumption, *n* (%) | 42 (39.3) | 10 (40.0) | 32 (39.0) | 1.000 |
| ApoE ε4 carrier, *n* (%) | 33 (30.8) | 7 (28.0) | 26 (31.7) | 0.809 |
| ***Comprehensive geriatric assessment*** | | | | |
| Barthel index | 100 | 100, 98–100 | 100 | 0.298 |
| IADL impairment, *n* (%) | 48 (44.9) | 12 (48.0) | 36 (43.9) | 0.819 |
| DBDS | 8, 4–14 | 6, 3–15 | 10, 4–14 | 0.470 |
| GDS* | 2, 1–5 | 4, 2–6 | 2, 1–4 | 0.013 |
| Vitality index | 10, 9–10 | 10, 9–10 | 10, 9–10 | 0.339 |
| ZBI | 11, 3–22 | 12, 3–23 | 10.5, 4–22 | 0.953 |
| MNA-SF | 12, 11–13 | 12, 11–13 | 13, 11–13 | 0.497 |
| ***Cognitive function*** | | | | |
| MMSE score | 24, 21–28 | 26, 19–29 | 24, 21–28 | 0.994 |
| CDR-GB |  |  |  | 0.478 |
| 0, *n* (%) | 21 (19.6) | 7 (28.0) | 14 (17.1) |  |
| 0.5, *n* (%) | 72 (67.3) | 14 (56.0) | 58 (70.7) |  |
| 1, *n* (%) | 13 (12.1) | 4 (16.0) | 9 (11.0) |  |
| 2, *n* (%) | 0 (0) | 0 (0) | 0 (0) |  |
| 3, *n* (%) | 1 (1.0) | 0 (0) | 1 (1.2) |  |
| CDR-SB | 2.0, 0.5–3.5 | 2.0, 0.5–4.0 | 2.0, 0.5–3.5 | 0.793 |
| ADAS-cog | 9.0, 5.7–14 | 8.0, 4.7–15 | 9.3, 5.7–14 | 0.645 |
| RCPM | 28, 24–32 | 30, 23–34 | 28, 24–31 | 0.354 |
| FAB | 11, 9–13 | 12, 9–14 | 11, 9–13 | 0.803 |
| LM-WMSR I | 8, 4–15 | 10, 5–15 | 8, 4–15 | 0.495 |
| LM-WMSR II | 3, 0–8 | 3, 0–8 | 3, 0–8 | 0.595 |
| ***MRI findings*** | | | | |
| SLI, *n* (%) | 11 (10.3) | 4 (16.0) | 7 (8.5) | 0.279 |
| WMH, *n* (%) | 29 (27.1) | 6 (24.0) | 23 (28.1) | 0.801 |
| CMBs, *n* (%) | 23 (21.5) | 3 (12.0) | 20 (24.4) | 0.268 |
| CSS, *n* (%) | 7 (6.5) | 1 (4.0) | 6 (7.3) | 1.000 |
| VSRAD | 1.01, 0.65–2.03 | 0.88, 0.55–2.11 | 1.05, 0.65–2.03 | 0.524 |
| ***Blood flow reduction on SPECT images*** | | | | |
| Posterior cingulate gyrus and/or precuneus, *n* (%) | 72 (71.3) | 13 (56.5) | 59 (75.6) | 0.114 |
| ***Gut microbiota*** |  |  |  |  |
| Enterotype |  |  |  | 0.435 |
| Enterotype I, *n* (%) | 43 (40.2) | 10 (40.0) | 33 (40.2) |  |
| Enterotype II, *n* (%) | 5 (4.7) | 0 (0) | 5 (6.1) |  |
| Enterotype III, *n* (%) | 59 (55.1) | 15 (60) | 44 (53.7) |  |
| F/B ratio | 1.50, 0.81–2.31 | 1.56, 1.18–2.73 | 1.41, 0.75–2.29 | 0.263 |
| ***Metabolite*** * |  |  |  |  |
| Ammonia, mg/g | 0.69, 0.46–1.01 | 0.64, 0.50–0.81 | 0.78, 0.45–1.14 | 0.208 |
| Succinic acid, mg/g | 0.03, 0.03–0.41 | 0.03, 0.03–0.37 | 0.03, 0.03–0.44 | 0.438 |
| Lactic acid, mg/g | 0.03, 0.03–0.41 | 0.03, 0.03–10.2 | 0.03, 0.03–0.20 | 0.729 |
| Formic acid, mg/g | 0.05, 0.05–0.05 | 0.05, 0.05–0.05 | 0.05, 0.05–0.05 | 0.573 |
| Acetic acid, mg/g | 3.63, 1.47–7.64 | 2.52, 1.19–10.48 | 3.97, 1.45–7.64 | 0.514 |
| Propionic acid, mg/g | 0.83, 0.03–2.01 | 0.56, 0.03–1.73 | 0.88, 0.03–2.16 | 0.609 |
| Iso-butyric acid, mg/g | 0.11, 0.03–0.22 | 0.11, 0.03–0.18 | 0.11, 0.03–0.25 | 0.901 |
| n-butyric acid, mg/g | 0.30, 0.03–0.86 | 0.21, 0.03–0.72 | 0.32, 0.03–0.90 | 0.560 |
| Iso-valeric acid, mg/g | 0.13, 0.03–0.34 | 0.03, 0.03–0.32 | 0.14, 0.03–0.36 | 0.634 |
| n-valeric acid, mg/g | 0.43, 0.12–2.65 | 0.30, 0.12–2.13 | 0.44, 0.13–2.90 | 0.626 |
| Phenol, μg/g | 1.14, 0.60–2.11 | 1.04, 0.32–4.03 | 1.22, 0.59–2.05 | 0.800 |
| P-cresol, μg/g | 4.21, 0.15–118.07 | 1.34, 0.14–110.5 | 4.93, 0.15–119.4 | 0.779 |
| 4-Ethylphenoll, μg/g | 0.36, 0.001–1.01 | 0.16, 0.001–0.75 | 0.56, 0.001–1.12 | 0.209 |
| Indolel, μg/g | 6.04, 0.24–30.43 | 1.19, 0.20–27.4 | 7.5, 0.25–32.0 | 0.220 |
| Skatolel, μg/g | 0.001, 0.001–4.14 | 0.10, 0.001–9.62 | 0.001, 0.001–3.90 | 0.523 |

Data are represented as the median (interquartile range) or number of patients (%). Wilcoxon signed-rank and χ^2^ tests were used.

The asterisk indicates statistical significance (*P* < 0.05).

Abbreviations: CKD, chronic kidney disease; IHD, ischemic heart disease; ApoE, apolipoprotein E; IADL, instrumental activities of daily living; DBDS, Dementia Behaviour Disturbance Scale; GDS, Geriatric Depression Scale; ZBI, Zarit Caregiver Burden Interview; MNA-SF, Mini-Nutritional Assessment-Short Form; MMSE, Mini-Mental State Examination; CDR-GB, Clinical Dementia Rating Global Score; CDR-SB, Clinical Dementia Rating-Sum of Boxes; ADAS-cog, Alzheimer’s Disease Assessment Scale-Cognitive Subscale; RCPM, Raven’s Coloured Progressive Matrices; FAB, Frontal Assessment Battery; LM-WMSR, Logical Memory subtests I and II of the Wechsler Memory Scale-Revised; SLI, silent lacunar infarct; WMH, white matter hyperintensity; CMB, cerebral microbleeds; CSS, cortical superficial siderosis; VSRAD, voxel-based specific regional analysis system for Alzheimer’s disease; SPECT, single photon emission computed tomography. Enterotype I: Bacteroides >30%, Enterotype II: Prevotella >15%, Enterotype III: others.

**Table S3. Univariable logistic regression analysis of standardised metabolites in faecal samples and the relationship with dementia presence**

| Every 1 SD increment | OR | 95% CI | *P* |
| --- | --- | --- | --- |
| ***Metabolite*** |  |  |  |
| st-Ammonia, mg/g* | 1.6 | 1.0–2.5 | 0.033 |
| st-Succinic acid, mg/g | 1.0 | 0.4–1.8 | 0.987 |
| st-Lactic acid, mg/g* | 0.3 | 0.02–1.0 | 0.048 |
| st-Formic acid, mg/g | NA |  |  |
| st-Acetic acid, mg/g | 0.8 | 0.4–1.4 | 0.516 |
| st-Propionic acid, mg/g | 0.8 | 0.3–1.4 | 0.572 |
| st-Iso-butyric acid, mg/g | 0.9 | 0.2–1.5 | 0.737 |
| st-n-butyric acid, mg/g | 1.3 | 0.9–2.1 | 0.200 |
| st-Iso-valeric acid, mg/g* | 1.5 | 1.0–2.3 | 0.048 |
| st-n-valeric acid, mg/g | 0.7 | 0.3–1.1 | 0.143 |
| st-Phenol, μg/g | 1.3 | 0.9–2.1 | 0.197 |
| st-P-cresol, μg/g* | 1.6 | 1.0–2.5 | 0.038 |
| st-4-Ethylphenoll, μg/g | 1.0 | 0.5–1.7 | 0.876 |
| st-Indolel, μg/g | 1.4 | 0.9–2.2 | 0.101 |
| st-Skatolel, μg/g | 1.1 | 0.6–1.9 | 0.684 |

The dependent variable was the prevalence of dementia.

Asterisks indicate statistical significance (*P* < 0.05).

Abbreviations: SD; standard deviation; OR, odds ratio; CI, confidence interval; st-, standardised; NA, not available because of undetectable metabolite.

| **Table S4. Comparison between medication and metabolites** | | | | |
| --- | --- | --- | --- | --- |
| ***Metabolite*** * | ***Medication type*** |  |  |  |
|  |  | Medication group | No-medication group | *P* |
| Lactic acid, mg/g* | Anti-thrombotic drug | 0.06, 0.03–40.1 | 0.03, 0.03–0.11 | 0.028 |
| Acetic acid, mg/g* | Anti-thrombotic drug | 4.58, 3.58–14.6 | 3.15, 0.99–7.13 | 0.047 |
| Iso-valeric acid, mg/g* | Anti-thrombotic drug | 0.72, 0.24–3.92 | 0.31, 0.12–2.30 | 0.048 |
| n-butyric acid, mg/g* | Statin | 0.36, 0.11–1.17 | 0.15, 0.03–0.69 | 0.044 |
| Iso-valeric acid, mg/g* | Anti-hyperglycaemic drug | 0.35, 0.08–0.52 | 0.03, 0.03–0.29 | 0.043 |
| Phenol, μg/g* | Aperient | 0.33, 0.001–0.99 | 1.30, 0.70–2.20 | 0.020 |
| 4-Ethylphenoll, μg/g* | Aperient | 0.001, 0.001–0.49 | 1.12, 0.001–1.12 | 0.034 |
| Indolel, μg/g* | Aperient | 0.67, 0.001–10.4 | 7.37, 0.25–32.0 | 0.040 |

Data are represented as the median (interquartile range).

Wilcoxon signed-rank tests were used.

Asterisks indicate statistical significance (*P* < 0.05).

**Table S5. Multivariable logistic regression analysis of standardised ammonia and lactic acid concentrations and the relationship with dementia presence**

|  | OR | 95% CI | *P* |
| --- | --- | --- | --- |
| ***Model 1*** | | | |
| st-Ammonia* | 1.7 | 1.07–2.76 | 0.023 |
| st-Lactic acid | 0.3 | 0.02–1.01 | 0.051 |
| st-Age, years | 1.2 | 0.68–2.18 | 0.540 |
| Female sex* | 4.9 | 1.58–18.24 | 0.005 |
| st-Education year | 1.6 | 0.88–3.15 | 0.120 |
| ***Model 2*** | | | |
| st-Ammonia* | 1.8 | 1.09–3.00 | 0.021 |
| st-Lactic acid | 0.3 | 0.02–1.03 | 0.058 |
| st-Age, years | 1.2 | 0.65–2.34 | 0.574 |
| Female sex* | 4.0 | 1.19–15.65 | 0.024 |
| st-Education year | 1.7 | 0.88–3.32 | 0.118 |
| ApoE ε4 carrier* | 5.6 | 1.98–17.18 | 0.001 |
| ***Model 3*** | | | |
| st-Ammonia* | 1.8 | 1.08–3.14 | 0.023 |
| st-Lactic acid | 0.4 | 0.02–1.21 | 0.125 |
| st-Age, years | 1.1 | 0.58–2.36 | 0.728 |
| st-Female sex* | 4.5 | 1.26–19.06 | 0.020 |
| st-Education year | 1.8 | 0.91–3.76 | 0.091 |
| ApoE ε4 carrier* | 7.1 | 2.32–24.22 | <0.001 |
| Enterotype I | 0.5 | 0.11–1.77 | 0.258 |
| F/B ratio | 1.2 | 0.96–1.49 | 0.124 |
| ***Model 4*** | | | |
| st-Ammonia* | 1.8 | 1.05–3.15 | 0.031 |
| st-Lactic acid | 0.4 | 0.02–1.27 | 0.154 |
| st-Age, years | 1.3 | 0.63–2.81 | 0.498 |
| Female sex* | 4.6 | 1.28–20.36 | 0.019 |
| st-Education year | 1.9 | 0.93–3.93 | 0.079 |
| ApoE ε4 carrier* | 7.0 | 2.24–24.5 | <0.001 |
| Enterotype III | 3.6 | 0.89–16.01 | 0.073 |
| F/B ratio | 1.1 | 0.91–1.43 | 0.270 |

The dependent variable was the prevalence of dementia.

Asterisks indicate statistical significance (*P* < 0.05).

Model 1: adjusted for age, sex, years of education.

Model 2: adjusted for model 1 and the presence of ApoE ε4.

Model 3: adjusted for model 2, enterotype I, and F/B ratio.

Model 4: adjusted for model 2, enterotype III, and F/B ratio.

Abbreviations: CI, confidence interval; OR, odds ratio; st-, standardised; F/B ratio, Firmicutes/Bacteroidetes ratio.

**Table S6. Univariable and multivariable logistic regression analysis of standardised ammonia concentration and the relationship with dementia presence**

| st-Ammonia | OR | 95% CI | *P* |
| --- | --- | --- | --- |
| Univariable* | 1.6 | 1.04–2.52 | 0.033 |
| Model 1^a^* | 1.7 | 1.06–2.73 | 0.027 |
| Model 2^b^* | 1.8 | 1.07–2.95 | 0.025 |
| Model 3^c^* | 1.8 | 1.09–3.14 | 0.021 |
| Model 4^d^* | 2.0 | 1.03–4.14 | 0.040 |
| Model 5-1^e^* | 1.8 | 1.05–3.08 | 0.031 |
| Model 5-2^f^* | 1.7 | 1.03–3.10 | 0.039 |
| Model 6-1^g^* | 2.0 | 1.17–3.80 | 0.011 |
| Model 6-2^h^* | 2.1 | 1.17–4.00 | 0.012 |
| Model 7-1^i^* | 2.1 | 1.07–4.41 | 0.030 |
| Model 7-2^j^* | 2.1 | 1.17–4.00 | 0.012 |

The dependent variable was the prevalence of dementia.

Asterisks indicate statistical significance (*P* < 0.05).

Models 1–4: adjusting without enterotypes.

Models 5­–7: adjusting with enterotypes (having either enterotype I or III).

^a^Model 1: adjusted for age, sex, years of education.

^b^Model 2: adjusted for model 1 and ApoE ε4.

^c^Model 3: stepwise adjusted for model 2 and the prevalence of risk factors (hypertension, diabetes mellitus, dyslipidaemia, chronic kidney disease, ischemic heart disease, history of stroke, smoking habit, and an alcohol drinking habit).

^d^Model 4: stepwise adjusted for model 3, MRI findings (the presence of silent lacunar infarcts, white matter hypersensitivity, cerebral microbleeds, cortical superficial siderosis, and VSRAD scores), and SPECT findings (the presence or absence of a reduction in blood flow in the posterior cingulate gyrus and/or precuneus).

^e^Model 5-1: adjusted for model 2, enterotype I, and F/B ratio.

^f^Model 5-2: adjusted for model 2, enterotype III, and F/B ratio.

^g^Model 6-1: stepwise adjusted for model 5-1 and the prevalence of risk factors.

^h^Model 6-2: stepwise adjusted for model 5-2 and the prevalence of risk factors.

^i^Model 7-1: stepwise adjusted for model 6-1, MRI findings, and SPECT findings.

^j^Model 7-2: stepwise adjusted for model 6-2, MRI findings, and SPECT findings.

Abbreviations: st-, standardised; CI, confidence interval; OR, odds ratio; MRI, magnetic resonance imaging; VSRAD, the voxel-based specific regional analysis system for Alzheimer’s disease; SPECT, single photon emission-computed tomography; F/B ratio, Firmicutes/Bacteroidetes ratio.

**Table S7. Univariable and multivariable logistic regression analysis of standardised lactic acid concentration and the relationship with dementia presence**

| st-Lactic acid | OR | 95% CI | *P* |
| --- | --- | --- | --- |
| Univariable* | 0.28 | 0.02–0.99 | 0.048 |
| Model 1^a^ | 0.30 | 0.02–1.04 | 0.060 |
| Model 2^b^ | 0.30 | 0.02–1.08 | 0.072 |
| Model 3^c^* | 0.19 | 0.01–0.76 | 0.013 |
| Model 4^d^* | 0.10 | 0.01–0.46 | 0.001 |
| Model 5-1^e^ | 0.40 | 0.02–1.32 | 0.172 |
| Model 5-2^f^ | 0.42 | 0.02–1.38 | 0.203 |
| Model 6-1^g^ | 0.33 | 0.02–1.00 | 0.051 |
| Model 6-2^h^ | 0.28 | 0.01–0.90 | 0.027 |
| Model 7-1^i^ | 0.18 | 0.01–1.10 | 0.069 |
| Model 7-2^j^ | 0.17 | 0.01–1.06 | 0.061 |

Asterisks indicate statistical significance (*P* < 0.05).

Models 1­–4: adjusting without enterotypes.

Models 5–7: adjusting with enterotypes (having either enterotype I or III).

^a^Model 1: adjusted for age, sex, years of education.

^b^Model 2: adjusted for model 1 and ApoE ε4.

^c^Model 3: stepwise adjusted for model 2 and the prevalence of risk factors (hypertension, diabetes mellitus, dyslipidaemia, chronic kidney disease, ischemic heart disease, history of stroke, smoking habit, and an alcohol drinking habit).

^d^Model 4: stepwise adjusted for model 3, MRI findings (the presence of silent lacunar infarcts, white matter hypersensitivity, cerebral microbleeds, cortical superficial siderosis, and VSRAD scores), and SPECT findings (the presence or absence of a reduction in blood flow in the posterior cingulate gyrus and/or precuneus).

^e^Model 5-1: adjusted for model 2, enterotype I, and F/B ratio.

^f^Model 5-2: adjusted for model 2, enterotype III, and F/B ratio.

^g^Model 6-1: stepwise adjusted for model 5-1 and the prevalence of risk factors.

^h^Model 6-2: stepwise adjusted for model 5-2 and the prevalence of risk factors.

^i^Model 7-1: stepwise adjusted for model 6-1, MRI findings, and SPECT findings.

^j^Model 7-2: stepwise adjusted for model 6-2, MRI findings, and SPECT findings.

Abbreviations: st-, standardised; CI, confidence interval; OR, odds ratio; MRI, magnetic resonance imaging; VSRAD, the voxel-based specific regional analysis system for Alzheimer’s disease; SPECT, single photon emission-computed tomography.
